# Crystal structure of [*N*,*N*-bis­(di­phenyl­phospho­ro­thio­yl)amidato-κ^2^ *S*,*S*′]bis­(tri­phenyl­phosphane-κ*P*)copper(I) di­chloro­methane monosolvate

**DOI:** 10.1107/S2056989017009380

**Published:** 2017-07-04

**Authors:** Tatsuya Nishi, Toshiaki Tsukuda, Michihiro Nishikawa, Taro Tsubomura

**Affiliations:** a Department of Materials and Life Science, Seikei University, 3-3-1 Kichijoji-kitamachi, Musashino-shi, Tokyo, Japan; b Faculty of Education and Human Science, University of Yamanashi, 4-4-37, Takeda, Kofu, Yamanashi, Japan

**Keywords:** crystal structure, copper(I) complex, diphosphane di­sulfide ligand, tri­phenyl­phosphane

## Abstract

The title compound, [Cu(C_24_H_20_NP_2_S_2_)(C_18_H_15_P)_2_]·CH_2_Cl_2_ or [Cu(dppaS_2_)(PPh_3_)_2_]·CH_2_Cl_2_, is a neutral mononuclear copper(I) complex bearing an *N*,*N*-bis­(di­phenyl­phospho­rothio­yl)amidate (dppaS_2_^−^) ligand and two tri­phenyl­phosphane ligands. The molecular structure shows that the two S atoms of the dppaS_2_^−^ ligand [Cu—S = 2.3462 (9) and 2.3484 (9) Å] and the two P atoms of the two tri­phenyl­phosphane ligands [Cu—P = 2.3167 (9) and 2.2969 (9) Å] coordinate to the copper(I) atom, resulting in a tetra­hedral coordination geometry. The crystallographically observed mol­ecular structure is compared to the results of DFT calculations.

## Abstract 2

The title compound, [Cu(dppaS_2_)(PPh_3_)_2_], is a neutral mononuclear copper(I) complex bearing an *N*,*N*-bis­(di­phenyl­phospho­rothio­yl)amidate (dppaS_2_^−^) ligand and two tri­phenyl­phosphane ligands. The structure of this complex was obtained by X-ray diffraction and supported by DFT calculations.

## Chemical context   

Copper(I) complexes have been studied actively because of the abundance of the metal ore and their inter­esting lumin­escent properties (Costa *et al.*, 2012 ▸). The most well-explored copper(I) complexes are those bearing nitro­gen and phospho­rus donor atoms, which display strong emission and long-lived lifetime of the excited states (Czerwieniec *et al.*, 2013 ▸). On the other hand, Cu^I^ complexes bearing sulfur donor ligands have not been well studied in this respect. Several years ago, we reported some emissive copper(I) complexes bearing diphosphane di­sulfide (Dairiki *et al.*, 2009 ▸). In addition, an inter­esting reaction was also reported in which a sulfur atom of the diphosphane di­sulfide ligand was transferred to another diphosphane ligand (Tsukuda *et al.*, 2012 ▸). We describe here the crystal structure of a neutral copper(I) complex [Cu(dppaS_2_)(PPh_3_)_2_] bearing the anionic diphos­phane di­sulfide ligand *N*,*N*-bis­(di­phenyl­phospho­rothio­yl)amidate (dppaS_2_^−^) and two tri­phenyl­phosphane ligands.
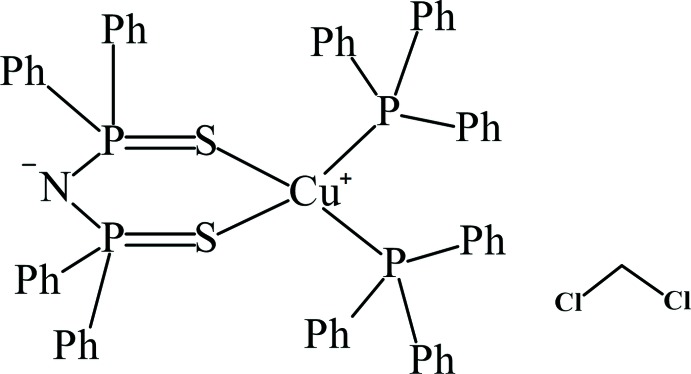


## Structural commentary   

**Figure 1:**
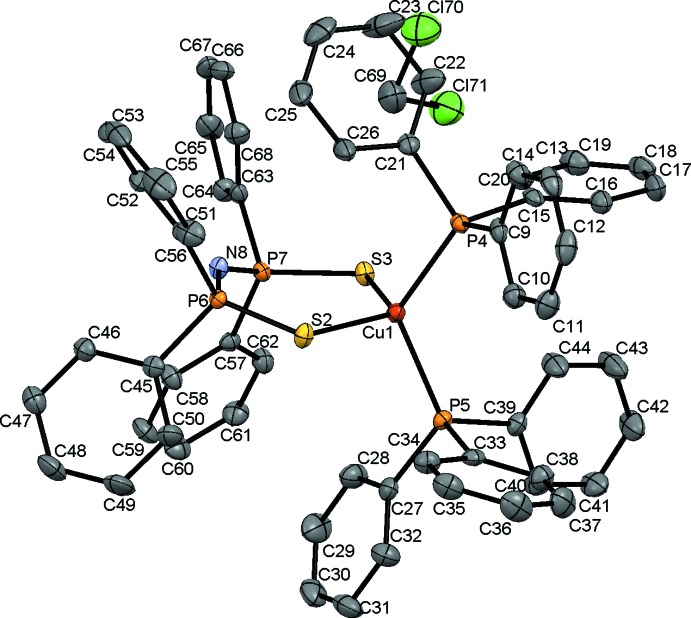
The structure of the molecular components of [Cu(dppaS_2_)(PPh_3_)_2_]·CH_2_Cl_2_, showing 50% probability displacement ellipsoids. H atoms have been omitted for clarity.

The molecular structure of [Cu(dppaS_2_)(PPh_3_)_2_] shows that the two sulfur atoms of the dppaS_2_^−^ ligand and the two phospho­rus atoms of the two tri­phenyl­phosphane ligands coordinate to the copper atom, resulting in a tetra­hedral coordination geometry (Fig. 1 ▸). The bond lengths between the copper(I) atom and the two sulfur atoms of the dppaS_2_^−^ ligand are Cu—S = 2.3462 (9) and 2.3484 (9) Å, and those between the copper atom and the phospho­rus atoms of the tri­phenyl­phosphane ligands are Cu—P = 2.3167 (9) and 2.2969 (9) Å. The diphosphine di­sulfide ligand forms a six-membered ring adopting a boat conformation. The bond order of the P6—S2 and P7—S3 bonds are considered to be slightly smaller than two because the lengths of the bonds are considerably longer than general P=S (= 1.91 Å) bond lengths (Wilson *et al.*, 1999 ▸) by *ca* 0.1 Å (Table 1 ▸). P6—N8 and P7—N8 appear to have double-bond character because at 1.587 (2) and 1.584 (2) Å, respectively, they are significantly shorter than the typical N—P (= 1.66 Å) bond length.

## Supra­molecular features   

**Figure 2:**
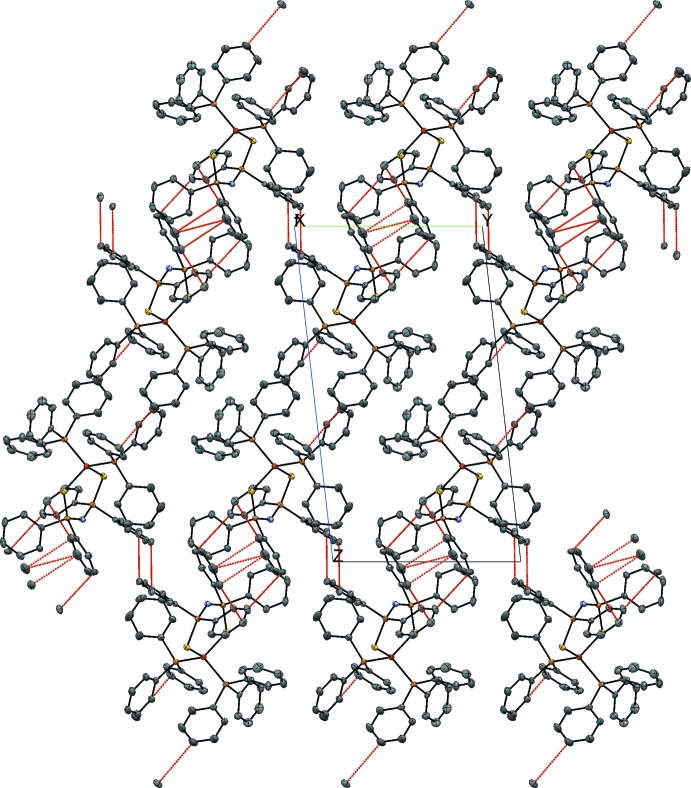
The crystal structure constructed from chains by C—H⋯C inter­actions (red dashed lines).

The space group of the crystal is *P* 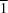, and the asymmetric unit consists of a complex mol­ecule, so that a unit cell contains two complex mol­ecules. In the crystal, weak C—H⋯C inter­actions are observed (Fig. 2 ▸ and Table 1 ▸).

## DFT calculations   

Calculations were performed with the *GAUSSIAN09* software (Frisch *et al.*, 2009 ▸) using the B3LYP method (Becke *et al.*, 1992 ▸, 1993 ▸; Lee *et al.*, 1988 ▸). The basis sets were as follows: copper, 6-311G with Wachters (1970 ▸) 4*p* functions; phos­phorus, oxygen, and nitro­gen, 6-31+G*; carbon, 6-31G*; and hydrogen, 6-31G. DFT calculations were performed and the results compared with experimental values (Table 2 ▸). The optimized structure in the singlet ground state is roughly consistent with crystal structure. The calculated Cu—S and Cu—P bond lengths are in agreement with the experimental value within 0.08 Å. The S—Cu—S and P—Cu—P bond angles, and the dihedral angle between the S/Cu/S and P/Cu/P planes obtained by DFT calculations and experimentally are in good agreement. The near right angle (87°) of the dihedral angle shows that the tetra­hedral geometry seems to be favorable for the complex.

## Synthesis and crystallization   

Under an argon atmosphere, 5 ml anhydrous di­chloro­methane were added with stirring to a mixture of *N*,*N*-bis­(di­phenyl­phospho­rothio­yl)amine (HdppaS_2_) (135 mg, 0.3 mmol) and potassium *tert*-butoxide (35 mg, 0.3 mmol). Tri­phenyl­phosphane (157.4 mg, 0.6 mmol) and [Cu(CH_3_CN)_4_]PF_6_ (111.8 mg, 0.3 mmol) were then added to the reaction solution. After the solution had been stirred for one h at room temperature, a white powder (KPF_6_) precipitated. The mixture was then filtered. The solution was added to ethanol (20 ml) and the resulting colorless crystals were obtained by filtration. Yield 256 mg (82%). Analysis found: C, 69.43; H, 4.85; N, 1.36%. Calculated for [Cu(dppaS_2_)(PPh_3_)_2_], C_60_H_50_NS_2_P_4_Cu: C, 69.51; H, 4.86; N, 1.35%. ^31^P{^1^H} NMR (202 MHz, CDCl_3_) 34.6 (*t*, *J* = 53.16 Hz, dppaS_2_), −2.5 (*s*, *br*, tri­phenyl­phosphane). Broadening of the ^31^P signals of the phospho­rus atoms directly coordinating to the copper atom, which has a large quadrupole moment, has frequently been observed (von Rekowski *et al.*, 2014 ▸). Single crystals suitable for X-ray diffraction were obtained during the synthetic procedure.

## Refinement   

Crystal data, data collection and structure refinement details are summarized in Table 3 ▸. The non-hydrogen atoms were refined anisotropically. All H atoms were positioned geometrically and refined isotropically using the riding model with C—H = 0.99 Å and *U*_iso_(H) = 1.2*U*_eq_(C) for methyl­ene groups, and 0.95 Å and *U*_iso_(H) = 1.2*U*_eq_(C) for aromatic groups.

## Supplementary Material

Crystal structure: contains datablock(s) global, I. DOI: 10.1107/S2056989017009380/im2478sup1.cif

Structure factors: contains datablock(s) I. DOI: 10.1107/S2056989017009380/im2478Isup2.hkl

Supporting information file. DOI: 10.1107/S2056989017009380/im2478Isup3.mol

CCDC reference: 1557968

Additional supporting information:  crystallographic information; 3D view; checkCIF report

## Figures and Tables

**Table 1:** Hydrogen-bond geometry (Å, °)

*D*—H⋯*A*	*D*—H	H⋯*A*	*D*⋯*A*	*D*—H⋯*A*
C42—H42⋯C13^i^	0.95	2.91	3.586 (5)	129
C59—H59⋯C53^ii^	0.95	2.79	3.590 (5)	142
C56—H56⋯C60^iii^	0.95	2.69	3.589 (6)	157
C66—H66⋯C65^iv^	0.95	3.02	3.468 (5)	111
C55—H55⋯C55^v^	0.95	3.71	3.552 (5)	73
C55—H55⋯C56^v^	0.95	3.47	3.495 (5)	83

Symmetry codes: (i) 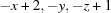; (ii) 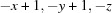; (iii) 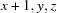; (iv) 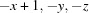; (v) 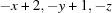.

**Table 2:** Bond lengths and angles (Å, °) for the optimized and obtained structure of [Cu(dppaS_2_)(PPh_3_)_2_]

Parameter	calculated (singlet)	crystal
Cu—S	2.422, 2.428	2.3462 (9), 2.3484 (9)
Cu—P	2.336, 2.329	2.2969 (9), 2.3167 (9)
S—P	2.042, 2.042	1.9920 (11), 2.0047 (11)
P—N	1.608, 1.607	1.587 (2), 1.584 (2)
S—Cu—S	111.75	114.96 (3)
P—Cu—P	122.81	121.28 (3)
Dihedral angle	87.74	86.69

The dihedral angle is between the S2/Cu1/S3 and P4/Cu1/P5 planes.

**Table 3:** Experimental details

Crystal data	
Chemical formula	[Cu(C_24_H_20_NP_2_S_2_)(C_18_H_15_P)_2_]·CH_2_Cl_2_
*M*_r_	1121.56
Crystal system, space group	Triclinic, *P* 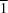
Temperature (K)	123
*a*, *b*, *c* (Å)	10.610 (3), 12.929 (3), 21.782 (5)
α, β, γ (°)	80.450 (8), 80.644 (8), 67.642 (7)
*V* (Å^3^)	2708.8 (12)
*Z*	2
Radiation type	Mo *K*α
μ (mm^−1^)	0.74
Crystal size (mm)	0.50 × 0.40 × 0.40

Data collection	
Diffractometer	Rigaku Saturn70
Absorption correction	Numerical (*NUMABS*; Rigaku, 1999 ▸)
*T*_min_, *T*_max_	0.821, 0.863
No. of measured, independent and observed [*F* ^2^ > 2.0σ(*F* ^2^)] reflections	24835, 11403, 9450
*R*_int_	0.042
(sin θ/λ)_max_ (Å^−1^)	0.649

Refinement	
*R*[*F* ^2^ > 2σ(*F* ^2^)], *wR*(*F* ^2^), *S*	0.049, 0.152, 0.82
No. of reflections	11403
No. of parameters	640
H-atom treatment	H-atom parameters constrained
Δρ_max_, Δρ_min_ (e Å^−3^)	1.10, −0.46

Computer programs: *CrystalClear* (Rigaku, 2000 ▸), *SIR92* (Altomare *et al.*, 1993 ▸), *SHELXL97* (Sheldrick, 2008 ▸), *Mercury* (Macrae *et al.*, 2006 ▸) and *CrystalStructure* (Rigaku, 2015 ▸).

## supplementary crystallographic information

### Crystal data

**Table:**

[Cu(C_24_H_20_NP_2_S_2_)(C_18_H_15_P)_2_]·CH_2_Cl_2_	*Z* = 2
*M**_r_* = 1121.56	*F*(000) = 1160.00
Triclinic, *P*1	*D*_x_ = 1.375 Mg m^−^^3^
*a* = 10.610 (3) Å	Mo *K*α radiation, λ = 0.71075 Å
*b* = 12.929 (3) Å	Cell parameters from 6125 reflections
*c* = 21.782 (5) Å	θ = 3.2–27.5°
α = 80.450 (8)°	µ = 0.74 mm^−^^1^
β = 80.644 (8)°	*T* = 123 K
γ = 67.642 (7)°	Prism, colorless
*V* = 2708.8 (12) Å^3^	0.50 × 0.40 × 0.40 mm

### Data collection

**Table:**

Rigaku Saturn70 diffractometer	9450 reflections with *F*^2^ > 2.0σ(*F*^2^)
Detector resolution: 29.257 pixels mm^-1^	*R*_int_ = 0.042
ω scans	θ_max_ = 27.5°, θ_min_ = 3.2°
Absorption correction: numerical (NUMABS; Rigaku, 1999)	*h* = −12→13
*T*_min_ = 0.821, *T*_max_ = 0.863	*k* = −16→16
24835 measured reflections	*l* = −28→28
11403 independent reflections	

### Refinement

**Table:**

Refinement on *F*^2^	Secondary atom site location: difference Fourier map
*R*[*F*^2^ > 2σ(*F*^2^)] = 0.049	Hydrogen site location: inferred from neighbouring sites
*wR*(*F*^2^) = 0.152	H-atom parameters constrained
*S* = 0.82	*w* = 1/[σ^2^(*F*_o_^2^) + (0.1058*P*)^2^ + 7.313*P*] where *P* = (*F*_o_^2^ + 2*F*_c_^2^)/3
11403 reflections	(Δ/σ)_max_ = 0.001
640 parameters	Δρ_max_ = 1.10 e Å^−^^3^
0 restraints	Δρ_min_ = −0.46 e Å^−^^3^
Primary atom site location: structure-invariant direct methods	

### Special details

**Table:**

Geometry. All esds (except the esd in the dihedral angle between two l.s. planes) are estimated using the full covariance matrix. The cell esds are taken into account individually in the estimation of esds in distances, angles and torsion angles; correlations between esds in cell parameters are only used when they are defined by crystal symmetry. An approximate (isotropic) treatment of cell esds is used for estimating esds involving l.s. planes.
Refinement. Refinement was performed using all reflections. The weighted R-factor (wR) and goodness of fit (S) are based on F^2^. R-factor (gt) are based on F. The threshold expression of F^2^ > 2.0 sigma(F^2^) is used only for calculating R-factor (gt).

### Fractional atomic coordinates and isotropic or equivalent isotropic displacement parameters (Å^2^)

**Table:**

	*x*	*y*	*z*	*U*_iso_*/*U*_eq_	
Cu1	0.76468 (4)	0.25233 (3)	0.284204 (15)	0.01466 (11)	
Cl70	0.85178 (13)	−0.23334 (11)	0.21386 (6)	0.0590 (3)	
Cl71	0.58013 (13)	−0.20717 (10)	0.27758 (5)	0.0551 (3)	
S2	0.80813 (8)	0.39124 (6)	0.20945 (3)	0.01882 (16)	
S3	0.62471 (8)	0.16942 (6)	0.25477 (3)	0.01728 (16)	
P4	0.96915 (8)	0.10274 (6)	0.29819 (3)	0.01554 (16)	
P5	0.64070 (8)	0.35492 (6)	0.36493 (3)	0.01494 (16)	
P6	0.74273 (7)	0.38722 (6)	0.12949 (3)	0.01317 (15)	
P7	0.57225 (7)	0.24508 (6)	0.16971 (3)	0.01315 (15)	
N8	0.6625 (3)	0.3073 (2)	0.12584 (11)	0.0159 (5)	
C9	1.1108 (3)	0.1251 (3)	0.32436 (13)	0.0190 (6)	
C10	1.0813 (3)	0.2140 (3)	0.35943 (14)	0.0229 (6)	
H10	0.9892	0.2639	0.3669	0.027*	
C11	1.1849 (4)	0.2305 (3)	0.38359 (16)	0.0287 (7)	
H11	1.1634	0.2920	0.4070	0.034*	
C12	1.3195 (4)	0.1574 (3)	0.37344 (16)	0.0302 (8)	
H12	1.3902	0.1683	0.3902	0.036*	
C13	1.3509 (3)	0.0681 (3)	0.33867 (16)	0.0278 (7)	
H13	1.4431	0.0180	0.3317	0.033*	
C14	1.2479 (3)	0.0519 (3)	0.31409 (15)	0.0239 (7)	
H14	1.2702	−0.0091	0.2902	0.029*	
C15	0.9526 (3)	−0.0154 (2)	0.35433 (14)	0.0189 (6)	
C16	1.0157 (3)	−0.0502 (3)	0.40937 (15)	0.0236 (7)	
H16	1.0770	−0.0173	0.4172	0.028*	
C17	0.9886 (4)	−0.1336 (3)	0.45308 (16)	0.0321 (8)	
H17	1.0306	−0.1564	0.4909	0.038*	
C18	0.9015 (4)	−0.1828 (3)	0.44165 (17)	0.0324 (8)	
H18	0.8839	−0.2396	0.4715	0.039*	
C19	0.8395 (4)	−0.1496 (3)	0.38663 (17)	0.0292 (7)	
H19	0.7804	−0.1841	0.3784	0.035*	
C20	0.8645 (3)	−0.0654 (3)	0.34365 (15)	0.0238 (7)	
H20	0.8205	−0.0417	0.3064	0.029*	
C21	1.0443 (3)	0.0385 (3)	0.22537 (14)	0.0195 (6)	
C22	1.0856 (5)	−0.0754 (3)	0.22047 (19)	0.0429 (10)	
H22	1.0819	−0.1262	0.2570	0.052*	
C23	1.1329 (5)	−0.1163 (4)	0.1620 (2)	0.0527 (13)	
H23	1.1600	−0.1945	0.1591	0.063*	
C24	1.1400 (4)	−0.0438 (4)	0.10906 (18)	0.0391 (9)	
H24	1.1696	−0.0713	0.0693	0.047*	
C25	1.1041 (4)	0.0690 (3)	0.11369 (16)	0.0368 (9)	
H25	1.1126	0.1188	0.0773	0.044*	
C26	1.0556 (4)	0.1104 (3)	0.17139 (15)	0.0292 (8)	
H26	1.0298	0.1886	0.1740	0.035*	
C27	0.4811 (3)	0.4736 (3)	0.35029 (13)	0.0196 (6)	
C28	0.3841 (4)	0.4530 (3)	0.32282 (17)	0.0310 (8)	
H28	0.4056	0.3815	0.3092	0.037*	
C29	0.2562 (4)	0.5366 (4)	0.31530 (19)	0.0416 (10)	
H29	0.1905	0.5219	0.2968	0.050*	
C30	0.2244 (4)	0.6412 (3)	0.33470 (18)	0.0371 (9)	
H30	0.1371	0.6982	0.3294	0.045*	
C31	0.3199 (4)	0.6625 (3)	0.36169 (18)	0.0343 (8)	
H31	0.2982	0.7345	0.3747	0.041*	
C32	0.4474 (4)	0.5792 (3)	0.36994 (17)	0.0271 (7)	
H32	0.5120	0.5941	0.3891	0.032*	
C33	0.7372 (3)	0.4213 (2)	0.39628 (14)	0.0179 (6)	
C34	0.7933 (3)	0.4906 (3)	0.35449 (15)	0.0226 (6)	
H34	0.7762	0.5051	0.3118	0.027*	
C35	0.8738 (3)	0.5382 (3)	0.37471 (17)	0.0280 (7)	
H35	0.9120	0.5851	0.3460	0.034*	
C36	0.8987 (4)	0.5173 (3)	0.43712 (19)	0.0340 (8)	
H36	0.9545	0.5497	0.4509	0.041*	
C37	0.8428 (4)	0.4497 (3)	0.47931 (18)	0.0329 (8)	
H37	0.8591	0.4366	0.5220	0.039*	
C38	0.7625 (3)	0.4009 (3)	0.45910 (15)	0.0244 (7)	
H38	0.7250	0.3537	0.4880	0.029*	
C39	0.5844 (3)	0.2747 (3)	0.43368 (13)	0.0182 (6)	
C40	0.4726 (4)	0.3246 (3)	0.47633 (15)	0.0248 (7)	
H40	0.4221	0.4032	0.4701	0.030*	
C41	0.4348 (4)	0.2593 (3)	0.52796 (16)	0.0308 (8)	
H41	0.3588	0.2938	0.5569	0.037*	
C42	0.5062 (4)	0.1453 (3)	0.53751 (17)	0.0356 (9)	
H42	0.4792	0.1013	0.5728	0.043*	
C43	0.6176 (4)	0.0947 (3)	0.49559 (19)	0.0437 (11)	
H43	0.6681	0.0161	0.5022	0.052*	
C44	0.6552 (4)	0.1599 (3)	0.44364 (17)	0.0323 (8)	
H44	0.7307	0.1249	0.4146	0.039*	
C45	0.6382 (3)	0.5305 (2)	0.10170 (14)	0.0169 (6)	
C46	0.6032 (3)	0.5538 (3)	0.04012 (14)	0.0204 (6)	
H46	0.6394	0.4961	0.0132	0.024*	
C47	0.5162 (3)	0.6605 (3)	0.01796 (16)	0.0252 (7)	
H47	0.4925	0.6752	−0.0237	0.030*	
C48	0.4647 (4)	0.7446 (3)	0.05696 (19)	0.0333 (8)	
H48	0.4068	0.8179	0.0419	0.040*	
C49	0.4970 (4)	0.7226 (3)	0.11759 (19)	0.0377 (9)	
H49	0.4600	0.7808	0.1442	0.045*	
C50	0.5829 (4)	0.6165 (3)	0.14057 (17)	0.0281 (7)	
H50	0.6040	0.6025	0.1826	0.034*	
C51	0.8854 (3)	0.3508 (2)	0.06807 (13)	0.0168 (6)	
C52	0.8794 (3)	0.2986 (3)	0.01793 (14)	0.0238 (7)	
H52	0.8032	0.2776	0.0170	0.029*	
C53	0.9859 (4)	0.2774 (3)	−0.03074 (15)	0.0325 (8)	
H53	0.9819	0.2422	−0.0650	0.039*	
C54	1.0976 (4)	0.3076 (3)	−0.02928 (17)	0.0380 (10)	
H54	1.1694	0.2938	−0.0627	0.046*	
C55	1.1041 (4)	0.3575 (3)	0.0205 (2)	0.0377 (9)	
H55	1.1815	0.3768	0.0218	0.045*	
C56	0.9978 (3)	0.3798 (3)	0.06922 (17)	0.0275 (7)	
H56	1.0025	0.4151	0.1033	0.033*	
C57	0.3936 (3)	0.3388 (2)	0.17572 (13)	0.0155 (5)	
C58	0.3555 (3)	0.4422 (3)	0.13879 (15)	0.0210 (6)	
H58	0.4230	0.4632	0.1112	0.025*	
C59	0.2185 (3)	0.5153 (3)	0.14200 (17)	0.0271 (7)	
H59	0.1934	0.5864	0.1173	0.033*	
C60	0.1201 (3)	0.4842 (3)	0.18100 (16)	0.0259 (7)	
H60	0.0268	0.5333	0.1826	0.031*	
C61	0.1564 (3)	0.3817 (3)	0.21796 (15)	0.0248 (7)	
H61	0.0880	0.3606	0.2447	0.030*	
C62	0.2930 (3)	0.3093 (3)	0.21607 (14)	0.0219 (6)	
H62	0.3178	0.2398	0.2423	0.026*	
C63	0.5829 (3)	0.1341 (2)	0.12530 (13)	0.0169 (6)	
C64	0.4714 (3)	0.1058 (3)	0.12004 (15)	0.0224 (6)	
H64	0.3829	0.1468	0.1390	0.027*	
C65	0.4902 (4)	0.0167 (3)	0.08673 (17)	0.0279 (7)	
H65	0.4141	−0.0027	0.0832	0.033*	
C66	0.6187 (4)	−0.0436 (3)	0.05883 (15)	0.0276 (7)	
H66	0.6307	−0.1041	0.0362	0.033*	
C67	0.7296 (4)	−0.0155 (3)	0.06404 (15)	0.0258 (7)	
H67	0.8179	−0.0569	0.0450	0.031*	
C68	0.7123 (3)	0.0732 (3)	0.09719 (15)	0.0230 (6)	
H68	0.7888	0.0922	0.1006	0.028*	
C69	0.6779 (5)	−0.1451 (4)	0.22122 (19)	0.0423 (10)	
H69A	0.6417	−0.1301	0.1802	0.051*	
H69B	0.6693	−0.0723	0.2337	0.051*	

### Atomic displacement parameters (Å^2^)

**Table:**

	*U*^11^	*U*^22^	*U*^33^	*U*^12^	*U*^13^	*U*^23^
Cu1	0.01456 (19)	0.01581 (19)	0.01294 (17)	−0.00524 (14)	−0.00092 (13)	−0.00102 (13)
Cl70	0.0466 (7)	0.0637 (8)	0.0562 (7)	−0.0086 (6)	0.0035 (5)	−0.0146 (6)
Cl71	0.0605 (7)	0.0490 (6)	0.0453 (6)	−0.0167 (6)	0.0111 (5)	−0.0010 (5)
S2	0.0220 (4)	0.0220 (4)	0.0151 (3)	−0.0117 (3)	−0.0030 (3)	0.0008 (3)
S3	0.0178 (4)	0.0185 (4)	0.0165 (3)	−0.0085 (3)	−0.0045 (3)	0.0024 (3)
P4	0.0140 (4)	0.0145 (4)	0.0153 (3)	−0.0038 (3)	−0.0005 (3)	0.0013 (3)
P5	0.0144 (4)	0.0161 (4)	0.0145 (3)	−0.0057 (3)	−0.0009 (3)	−0.0026 (3)
P6	0.0126 (4)	0.0137 (3)	0.0131 (3)	−0.0054 (3)	0.0000 (3)	−0.0008 (3)
P7	0.0126 (4)	0.0129 (3)	0.0140 (3)	−0.0050 (3)	−0.0022 (3)	0.0001 (3)
N8	0.0172 (13)	0.0176 (12)	0.0149 (11)	−0.0095 (10)	−0.0013 (9)	−0.0004 (9)
C9	0.0170 (15)	0.0226 (15)	0.0149 (13)	−0.0073 (12)	−0.0019 (11)	0.0054 (11)
C10	0.0222 (16)	0.0242 (16)	0.0213 (15)	−0.0083 (13)	−0.0022 (12)	−0.0001 (12)
C11	0.0308 (19)	0.0359 (19)	0.0242 (16)	−0.0174 (16)	−0.0040 (14)	−0.0024 (14)
C12	0.0264 (18)	0.046 (2)	0.0224 (16)	−0.0210 (16)	−0.0054 (13)	0.0074 (14)
C13	0.0153 (15)	0.0327 (19)	0.0278 (17)	−0.0049 (14)	−0.0036 (13)	0.0087 (14)
C14	0.0201 (16)	0.0226 (16)	0.0249 (16)	−0.0063 (13)	−0.0008 (12)	0.0030 (12)
C15	0.0152 (14)	0.0140 (14)	0.0195 (14)	0.0008 (11)	0.0027 (11)	0.0004 (11)
C16	0.0248 (17)	0.0205 (16)	0.0207 (15)	−0.0055 (13)	0.0007 (12)	0.0008 (12)
C17	0.043 (2)	0.0223 (17)	0.0215 (16)	−0.0051 (16)	−0.0021 (15)	0.0051 (13)
C18	0.040 (2)	0.0172 (16)	0.0297 (18)	−0.0066 (15)	0.0103 (15)	0.0023 (13)
C19	0.0287 (18)	0.0216 (16)	0.0349 (18)	−0.0106 (14)	0.0051 (14)	−0.0011 (14)
C20	0.0236 (17)	0.0197 (15)	0.0243 (16)	−0.0070 (13)	0.0031 (13)	−0.0003 (12)
C21	0.0144 (14)	0.0213 (15)	0.0193 (14)	−0.0044 (12)	0.0010 (11)	−0.0012 (11)
C22	0.064 (3)	0.0276 (19)	0.033 (2)	−0.022 (2)	0.0215 (19)	−0.0073 (15)
C23	0.076 (3)	0.039 (2)	0.046 (2)	−0.030 (2)	0.031 (2)	−0.0241 (19)
C24	0.032 (2)	0.056 (3)	0.0275 (18)	−0.0149 (19)	0.0105 (15)	−0.0174 (17)
C25	0.0292 (19)	0.041 (2)	0.0201 (16)	0.0060 (17)	0.0030 (14)	−0.0002 (15)
C26	0.0280 (18)	0.0241 (17)	0.0209 (16)	0.0020 (14)	0.0043 (13)	0.0021 (13)
C27	0.0220 (16)	0.0218 (15)	0.0142 (13)	−0.0083 (13)	−0.0002 (11)	−0.0012 (11)
C28	0.0263 (18)	0.0331 (19)	0.0322 (18)	−0.0027 (15)	−0.0099 (14)	−0.0132 (15)
C29	0.0240 (19)	0.052 (3)	0.042 (2)	0.0023 (17)	−0.0153 (16)	−0.0146 (19)
C30	0.0238 (18)	0.038 (2)	0.0337 (19)	0.0048 (16)	−0.0053 (15)	0.0009 (16)
C31	0.0298 (19)	0.0197 (17)	0.042 (2)	0.0004 (15)	0.0012 (16)	−0.0015 (15)
C32	0.0226 (17)	0.0215 (16)	0.0345 (18)	−0.0049 (14)	−0.0023 (14)	−0.0047 (13)
C33	0.0135 (14)	0.0161 (14)	0.0216 (14)	−0.0011 (11)	−0.0001 (11)	−0.0072 (11)
C34	0.0214 (16)	0.0221 (16)	0.0262 (16)	−0.0097 (13)	−0.0016 (12)	−0.0048 (12)
C35	0.0222 (17)	0.0239 (17)	0.0399 (19)	−0.0104 (14)	−0.0036 (14)	−0.0042 (14)
C36	0.0283 (19)	0.0327 (19)	0.049 (2)	−0.0145 (16)	−0.0147 (16)	−0.0067 (16)
C37	0.032 (2)	0.036 (2)	0.0338 (19)	−0.0117 (16)	−0.0156 (15)	−0.0034 (15)
C38	0.0227 (17)	0.0270 (17)	0.0244 (16)	−0.0088 (14)	−0.0041 (13)	−0.0045 (13)
C39	0.0173 (15)	0.0237 (15)	0.0149 (13)	−0.0093 (12)	−0.0008 (11)	−0.0027 (11)
C40	0.0272 (17)	0.0204 (16)	0.0235 (16)	−0.0081 (14)	0.0063 (13)	−0.0030 (12)
C41	0.0276 (18)	0.0298 (18)	0.0276 (17)	−0.0079 (15)	0.0105 (14)	−0.0021 (14)
C42	0.034 (2)	0.033 (2)	0.0280 (18)	−0.0074 (16)	0.0082 (15)	0.0081 (15)
C43	0.040 (2)	0.0253 (19)	0.040 (2)	0.0027 (17)	0.0132 (18)	0.0111 (16)
C44	0.0254 (18)	0.0275 (18)	0.0290 (18)	−0.0002 (15)	0.0089 (14)	0.0027 (14)
C45	0.0158 (14)	0.0163 (14)	0.0192 (14)	−0.0090 (12)	0.0027 (11)	−0.0005 (11)
C46	0.0200 (15)	0.0190 (15)	0.0210 (15)	−0.0081 (12)	0.0007 (12)	0.0006 (11)
C47	0.0211 (16)	0.0252 (17)	0.0270 (16)	−0.0082 (14)	−0.0046 (13)	0.0050 (13)
C48	0.033 (2)	0.0152 (16)	0.046 (2)	−0.0016 (14)	−0.0125 (16)	0.0019 (14)
C49	0.045 (2)	0.0158 (16)	0.047 (2)	0.0017 (16)	−0.0127 (18)	−0.0105 (15)
C50	0.0321 (19)	0.0205 (16)	0.0297 (17)	−0.0039 (14)	−0.0073 (14)	−0.0077 (13)
C51	0.0151 (14)	0.0156 (14)	0.0142 (13)	−0.0015 (11)	−0.0001 (11)	0.0019 (10)
C52	0.0195 (16)	0.0244 (16)	0.0201 (15)	0.0005 (13)	−0.0027 (12)	−0.0027 (12)
C53	0.0308 (19)	0.0308 (19)	0.0161 (15)	0.0091 (15)	0.0017 (13)	−0.0025 (13)
C54	0.0279 (19)	0.031 (2)	0.0281 (18)	0.0064 (16)	0.0151 (15)	0.0086 (15)
C55	0.0227 (18)	0.033 (2)	0.048 (2)	−0.0121 (16)	0.0128 (16)	0.0079 (17)
C56	0.0222 (17)	0.0258 (17)	0.0332 (18)	−0.0118 (14)	0.0052 (14)	−0.0002 (14)
C57	0.0145 (14)	0.0169 (14)	0.0159 (13)	−0.0059 (11)	−0.0015 (11)	−0.0042 (10)
C58	0.0160 (15)	0.0209 (15)	0.0251 (15)	−0.0067 (12)	−0.0048 (12)	0.0024 (12)
C59	0.0215 (17)	0.0193 (16)	0.0352 (18)	−0.0011 (13)	−0.0103 (14)	0.0026 (13)
C60	0.0141 (15)	0.0293 (18)	0.0315 (17)	−0.0012 (13)	−0.0067 (13)	−0.0076 (14)
C61	0.0170 (16)	0.0310 (18)	0.0249 (16)	−0.0078 (14)	0.0006 (12)	−0.0037 (13)
C62	0.0201 (16)	0.0236 (16)	0.0206 (14)	−0.0081 (13)	−0.0029 (12)	0.0022 (12)
C63	0.0223 (15)	0.0134 (13)	0.0153 (13)	−0.0068 (12)	−0.0051 (11)	0.0016 (10)
C64	0.0226 (16)	0.0213 (16)	0.0258 (16)	−0.0090 (13)	−0.0060 (13)	−0.0035 (12)
C65	0.0320 (19)	0.0222 (16)	0.0360 (18)	−0.0121 (14)	−0.0142 (15)	−0.0051 (14)
C66	0.043 (2)	0.0187 (15)	0.0220 (15)	−0.0093 (15)	−0.0088 (14)	−0.0048 (12)
C67	0.0293 (18)	0.0166 (15)	0.0246 (16)	−0.0016 (13)	0.0025 (13)	−0.0050 (12)
C68	0.0240 (17)	0.0208 (15)	0.0251 (15)	−0.0101 (13)	0.0002 (13)	−0.0033 (12)
C69	0.052 (3)	0.037 (2)	0.035 (2)	−0.0132 (19)	−0.0062 (18)	0.0016 (16)

### Geometric parameters (Å, º)

**Table:**

C10—H10	0.9500	C46—H46	0.9500
C10—C11	1.391 (5)	C46—C47	1.394 (4)
C11—H11	0.9500	C47—H47	0.9500
C11—C12	1.387 (5)	C47—C48	1.381 (5)
C12—H12	0.9500	C48—H48	0.9500
C12—C13	1.391 (5)	C48—C49	1.375 (5)
C13—H13	0.9500	C49—H49	0.9500
C13—C14	1.388 (5)	C49—C50	1.390 (5)
C14—H14	0.9500	C50—H50	0.9500
C15—C20	1.389 (5)	C51—C56	1.384 (4)
C15—C16	1.393 (4)	C51—C52	1.397 (4)
C16—H16	0.9500	C52—H52	0.9500
C16—C17	1.399 (5)	C52—C53	1.395 (5)
C17—H17	0.9500	C53—H53	0.9500
C17—C18	1.377 (6)	C53—C54	1.387 (6)
C18—H18	0.9500	C54—H54	0.9500
C18—C19	1.387 (5)	C54—C55	1.374 (6)
C19—H19	0.9500	C55—H55	0.9500
C19—C20	1.390 (4)	C55—C56	1.395 (5)
C20—H20	0.9500	C56—H56	0.9500
C21—C26	1.392 (4)	C57—C62	1.396 (4)
C21—C22	1.385 (5)	C57—C58	1.391 (4)
C22—H22	0.9500	C58—H58	0.9500
C22—C23	1.403 (5)	C58—C59	1.398 (4)
C23—H23	0.9500	C59—H59	0.9500
C23—C24	1.372 (6)	C59—C60	1.375 (5)
C24—H24	0.9500	C60—H60	0.9500
C24—C25	1.377 (6)	C60—C61	1.382 (5)
C25—H25	0.9500	C61—H61	0.9500
C25—C26	1.391 (5)	C61—C62	1.392 (5)
C26—H26	0.9500	C62—H62	0.9500
C27—C32	1.396 (4)	C63—C68	1.395 (4)
C27—C28	1.396 (5)	C63—C64	1.390 (4)
C28—H28	0.9500	C64—H64	0.9500
C28—C29	1.391 (5)	C64—C65	1.396 (4)
C29—H29	0.9500	C65—H65	0.9500
C29—C30	1.385 (6)	C65—C66	1.384 (5)
C30—H30	0.9500	C66—H66	0.9500
C30—C31	1.381 (6)	C66—C67	1.382 (5)
C31—H31	0.9500	C67—H67	0.9500
C31—C32	1.389 (5)	C67—C68	1.394 (4)
C32—H32	0.9500	C68—H68	0.9500
C33—C38	1.400 (4)	C69—H69B	0.9900
C33—C34	1.394 (4)	C69—H69A	0.9900
C34—H34	0.9500	C9—C14	1.407 (4)
C34—C35	1.384 (4)	C9—C10	1.393 (5)
C35—H35	0.9500	Cl70—C69	1.759 (5)
C35—C36	1.390 (5)	Cl71—C69	1.766 (4)
C36—H36	0.9500	Cu1—S3	2.3484 (9)
C36—C37	1.382 (5)	Cu1—S2	2.3462 (9)
C37—H37	0.9500	Cu1—P5	2.2969 (9)
C37—C38	1.393 (5)	Cu1—P4	2.3167 (9)
C38—H38	0.9500	P4—C9	1.826 (3)
C39—C44	1.382 (5)	P4—C21	1.834 (3)
C39—C40	1.395 (4)	P4—C15	1.837 (3)
C40—H40	0.9500	P5—C39	1.828 (3)
C40—C41	1.391 (5)	P5—C33	1.828 (3)
C41—H41	0.9500	P5—C27	1.833 (3)
C41—C42	1.375 (5)	P6—N8	1.587 (2)
C42—H42	0.9500	P6—C51	1.819 (3)
C42—C43	1.385 (5)	P6—C45	1.820 (3)
C43—H43	0.9500	P7—N8	1.584 (2)
C43—C44	1.394 (5)	P7—C63	1.819 (3)
C44—H44	0.9500	P7—C57	1.817 (3)
C45—C50	1.398 (4)	S2—P6	1.9920 (11)
C45—C46	1.404 (4)	S3—P7	2.0047 (11)
			
P5—Cu1—P4	121.28 (3)	C33—C34—H34	119.8
P5—Cu1—S2	101.86 (3)	C34—C35—C36	119.9 (3)
P4—Cu1—S2	108.95 (3)	C34—C35—H35	120.0
P5—Cu1—S3	106.70 (3)	C36—C35—H35	120.0
P4—Cu1—S3	103.66 (4)	C37—C36—C35	120.4 (3)
S2—Cu1—S3	114.96 (3)	C37—C36—H36	119.8
P6—S2—Cu1	108.57 (4)	C35—C36—H36	119.8
P7—S3—Cu1	106.93 (4)	C36—C37—C38	119.9 (3)
C9—P4—C21	103.84 (14)	C36—C37—H37	120.0
C9—P4—C15	102.13 (14)	C38—C37—H37	120.0
C21—P4—C15	103.42 (14)	C37—C38—C33	120.0 (3)
C9—P4—Cu1	120.01 (11)	C37—C38—H38	120.0
C21—P4—Cu1	111.08 (10)	C33—C38—H38	120.0
C15—P4—Cu1	114.55 (10)	C44—C39—C40	118.7 (3)
C39—P5—C33	103.84 (14)	C44—C39—P5	118.7 (2)
C39—P5—C27	101.77 (14)	C40—C39—P5	122.6 (2)
C33—P5—C27	102.10 (14)	C39—C40—C41	120.1 (3)
C39—P5—Cu1	115.34 (10)	C39—C40—H40	120.0
C33—P5—Cu1	112.80 (10)	C41—C40—H40	120.0
C27—P5—Cu1	119.00 (10)	C42—C41—C40	120.7 (3)
N8—P6—C51	104.80 (13)	C42—C41—H41	119.6
N8—P6—C45	108.33 (13)	C40—C41—H41	119.6
C51—P6—C45	103.04 (13)	C41—C42—C43	119.8 (3)
N8—P6—S2	120.87 (10)	C41—C42—H42	120.1
C51—P6—S2	110.49 (10)	C43—C42—H42	120.1
C45—P6—S2	107.88 (10)	C44—C43—C42	119.5 (3)
N8—P7—C57	109.55 (13)	C44—C43—H43	120.2
N8—P7—C63	103.64 (13)	C42—C43—H43	120.2
C57—P7—C63	106.11 (13)	C39—C44—C43	121.2 (3)
N8—P7—S3	120.38 (10)	C39—C44—H44	119.4
C57—P7—S3	109.96 (10)	C43—C44—H44	119.4
C63—P7—S3	106.06 (10)	C50—C45—C46	118.5 (3)
P7—N8—P6	140.30 (16)	C50—C45—P6	122.6 (2)
C10—C9—C14	118.6 (3)	C46—C45—P6	118.8 (2)
C10—C9—P4	118.7 (2)	C47—C46—C45	120.9 (3)
C14—C9—P4	122.6 (2)	C47—C46—H46	119.5
C11—C10—C9	120.9 (3)	C45—C46—H46	119.5
C11—C10—H10	119.5	C48—C47—C46	119.5 (3)
C9—C10—H10	119.5	C48—C47—H47	120.2
C10—C11—C12	120.0 (3)	C46—C47—H47	120.2
C10—C11—H11	120.0	C49—C48—C47	120.2 (3)
C12—C11—H11	120.0	C49—C48—H48	119.9
C13—C12—C11	119.9 (3)	C47—C48—H48	119.9
C13—C12—H12	120.1	C48—C49—C50	121.1 (3)
C11—C12—H12	120.1	C48—C49—H49	119.5
C12—C13—C14	120.3 (3)	C50—C49—H49	119.5
C12—C13—H13	119.9	C49—C50—C45	119.9 (3)
C14—C13—H13	119.9	C49—C50—H50	120.1
C13—C14—C9	120.4 (3)	C45—C50—H50	120.1
C13—C14—H14	119.8	C56—C51—C52	119.5 (3)
C9—C14—H14	119.8	C56—C51—P6	120.0 (2)
C20—C15—C16	119.0 (3)	C52—C51—P6	120.4 (2)
C20—C15—P4	118.1 (2)	C53—C52—C51	119.7 (3)
C16—C15—P4	122.7 (2)	C53—C52—H52	120.1
C15—C16—C17	119.9 (3)	C51—C52—H52	120.1
C15—C16—H16	120.1	C54—C53—C52	120.2 (3)
C17—C16—H16	120.1	C54—C53—H53	119.9
C18—C17—C16	120.4 (3)	C52—C53—H53	119.9
C18—C17—H17	119.8	C55—C54—C53	120.0 (3)
C16—C17—H17	119.8	C55—C54—H54	120.0
C17—C18—C19	120.0 (3)	C53—C54—H54	120.0
C17—C18—H18	120.0	C54—C55—C56	120.3 (4)
C19—C18—H18	120.0	C54—C55—H55	119.8
C18—C19—C20	119.6 (3)	C56—C55—H55	119.8
C18—C19—H19	120.2	C51—C56—C55	120.2 (3)
C20—C19—H19	120.2	C51—C56—H56	119.9
C15—C20—C19	121.0 (3)	C55—C56—H56	119.9
C15—C20—H20	119.5	C62—C57—C58	119.0 (3)
C19—C20—H20	119.5	C62—C57—P7	121.7 (2)
C22—C21—C26	118.5 (3)	C58—C57—P7	119.3 (2)
C22—C21—P4	124.3 (2)	C57—C58—C59	120.5 (3)
C26—C21—P4	117.2 (2)	C57—C58—H58	119.8
C21—C22—C23	120.5 (4)	C59—C58—H58	119.8
C21—C22—H22	119.7	C60—C59—C58	119.9 (3)
C23—C22—H22	119.7	C60—C59—H59	120.0
C24—C23—C22	120.2 (4)	C58—C59—H59	120.0
C24—C23—H23	119.9	C59—C60—C61	120.2 (3)
C22—C23—H23	119.9	C59—C60—H60	119.9
C23—C24—C25	119.8 (3)	C61—C60—H60	119.9
C23—C24—H24	120.1	C60—C61—C62	120.2 (3)
C25—C24—H24	120.1	C60—C61—H61	119.9
C24—C25—C26	120.3 (3)	C62—C61—H61	119.9
C24—C25—H25	119.8	C57—C62—C61	120.1 (3)
C26—C25—H25	119.8	C57—C62—H62	119.9
C25—C26—C21	120.7 (3)	C61—C62—H62	119.9
C25—C26—H26	119.7	C64—C63—C68	119.6 (3)
C21—C26—H26	119.7	C64—C63—P7	123.7 (2)
C32—C27—C28	118.8 (3)	C68—C63—P7	116.7 (2)
C32—C27—P5	123.5 (2)	C63—C64—C65	119.7 (3)
C28—C27—P5	117.4 (2)	C63—C64—H64	120.1
C29—C28—C27	120.4 (3)	C65—C64—H64	120.1
C29—C28—H28	119.8	C66—C65—C64	120.6 (3)
C27—C28—H28	119.8	C66—C65—H65	119.7
C28—C29—C30	120.2 (4)	C64—C65—H65	119.7
C28—C29—H29	119.9	C67—C66—C65	119.8 (3)
C30—C29—H29	119.9	C67—C66—H66	120.1
C31—C30—C29	119.9 (3)	C65—C66—H66	120.1
C31—C30—H30	120.0	C66—C67—C68	120.3 (3)
C29—C30—H30	120.0	C66—C67—H67	119.9
C30—C31—C32	120.2 (3)	C68—C67—H67	119.9
C30—C31—H31	119.9	C67—C68—C63	120.1 (3)
C32—C31—H31	119.9	C67—C68—H68	120.0
C31—C32—C27	120.4 (3)	C63—C68—H68	120.0
C31—C32—H32	119.8	Cl70—C69—Cl71	111.2 (2)
C27—C32—H32	119.8	Cl70—C69—H69A	109.4
C34—C33—C38	119.3 (3)	Cl71—C69—H69A	109.4
C34—C33—P5	117.7 (2)	Cl70—C69—H69B	109.4
C38—C33—P5	122.9 (2)	Cl71—C69—H69B	109.4
C35—C34—C33	120.5 (3)	H69A—C69—H69B	108.0
C35—C34—H34	119.8		
			
P5—Cu1—S2—P6	−125.54 (4)	C29—C30—C31—C32	−0.5 (6)
P4—Cu1—S2—P6	105.18 (5)	C30—C31—C32—C27	0.9 (6)
S3—Cu1—S2—P6	−10.61 (5)	C28—C27—C32—C31	−0.6 (5)
P5—Cu1—S3—P7	110.80 (4)	P5—C27—C32—C31	−174.8 (3)
P4—Cu1—S3—P7	−120.10 (4)	C39—P5—C33—C34	−179.4 (2)
S2—Cu1—S3—P7	−1.31 (5)	C27—P5—C33—C34	−73.8 (3)
P5—Cu1—P4—C9	−58.34 (12)	Cu1—P5—C33—C34	55.1 (3)
S2—Cu1—P4—C9	59.23 (11)	C39—P5—C33—C38	4.1 (3)
S3—Cu1—P4—C9	−177.92 (11)	C27—P5—C33—C38	109.6 (3)
P5—Cu1—P4—C21	−179.58 (11)	Cu1—P5—C33—C38	−121.5 (2)
S2—Cu1—P4—C21	−62.01 (11)	C38—C33—C34—C35	0.3 (5)
S3—Cu1—P4—C21	60.85 (11)	P5—C33—C34—C35	−176.4 (2)
P5—Cu1—P4—C15	63.71 (12)	C33—C34—C35—C36	−0.2 (5)
S2—Cu1—P4—C15	−178.72 (11)	C34—C35—C36—C37	−0.4 (5)
S3—Cu1—P4—C15	−55.87 (12)	C35—C36—C37—C38	0.9 (6)
P4—Cu1—P5—C39	−59.55 (11)	C36—C37—C38—C33	−0.7 (5)
S2—Cu1—P5—C39	179.39 (11)	C34—C33—C38—C37	0.2 (5)
S3—Cu1—P5—C39	58.52 (11)	P5—C33—C38—C37	176.7 (3)
P4—Cu1—P5—C33	59.54 (11)	C33—P5—C39—C44	−101.2 (3)
S2—Cu1—P5—C33	−61.51 (11)	C27—P5—C39—C44	153.0 (3)
S3—Cu1—P5—C33	177.61 (11)	Cu1—P5—C39—C44	22.7 (3)
P4—Cu1—P5—C27	179.08 (11)	C33—P5—C39—C40	79.1 (3)
S2—Cu1—P5—C27	58.03 (12)	C27—P5—C39—C40	−26.7 (3)
S3—Cu1—P5—C27	−62.85 (12)	Cu1—P5—C39—C40	−156.9 (2)
Cu1—S2—P6—N8	5.53 (12)	C44—C39—C40—C41	0.6 (5)
Cu1—S2—P6—C51	−117.23 (10)	P5—C39—C40—C41	−179.8 (3)
Cu1—S2—P6—C45	130.84 (10)	C39—C40—C41—C42	−0.3 (6)
Cu1—S3—P7—N8	21.91 (12)	C40—C41—C42—C43	0.4 (6)
Cu1—S3—P7—C57	−106.82 (10)	C41—C42—C43—C44	−0.8 (7)
Cu1—S3—P7—C63	138.86 (11)	C40—C39—C44—C43	−0.9 (6)
C57—P7—N8—P6	87.7 (3)	P5—C39—C44—C43	179.4 (3)
C63—P7—N8—P6	−159.4 (3)	C42—C43—C44—C39	1.0 (7)
S3—P7—N8—P6	−41.2 (3)	N8—P6—C45—C50	114.7 (3)
C51—P6—N8—P7	149.0 (3)	C51—P6—C45—C50	−134.6 (3)
C45—P6—N8—P7	−101.5 (3)	S2—P6—C45—C50	−17.8 (3)
S2—P6—N8—P7	23.6 (3)	N8—P6—C45—C46	−60.5 (3)
C21—P4—C9—C10	151.0 (2)	C51—P6—C45—C46	50.2 (3)
C15—P4—C9—C10	−101.7 (3)	S2—P6—C45—C46	167.1 (2)
Cu1—P4—C9—C10	26.2 (3)	C50—C45—C46—C47	0.5 (5)
C21—P4—C9—C14	−33.4 (3)	P6—C45—C46—C47	175.8 (2)
C15—P4—C9—C14	73.9 (3)	C45—C46—C47—C48	0.6 (5)
Cu1—P4—C9—C14	−158.1 (2)	C46—C47—C48—C49	−1.2 (6)
C14—C9—C10—C11	0.3 (5)	C47—C48—C49—C50	0.8 (6)
P4—C9—C10—C11	176.2 (2)	C48—C49—C50—C45	0.2 (6)
C9—C10—C11—C12	−0.7 (5)	C46—C45—C50—C49	−0.9 (5)
C10—C11—C12—C13	0.5 (5)	P6—C45—C50—C49	−176.1 (3)
C11—C12—C13—C14	0.0 (5)	N8—P6—C51—C56	−161.3 (3)
C12—C13—C14—C9	−0.4 (5)	C45—P6—C51—C56	85.4 (3)
C10—C9—C14—C13	0.2 (4)	S2—P6—C51—C56	−29.6 (3)
P4—C9—C14—C13	−175.5 (2)	N8—P6—C51—C52	21.8 (3)
C9—P4—C15—C20	−171.8 (2)	C45—P6—C51—C52	−91.5 (3)
C21—P4—C15—C20	−64.2 (3)	S2—P6—C51—C52	153.5 (2)
Cu1—P4—C15—C20	56.8 (3)	C56—C51—C52—C53	−0.8 (5)
C9—P4—C15—C16	13.7 (3)	P6—C51—C52—C53	176.1 (2)
C21—P4—C15—C16	121.3 (3)	C51—C52—C53—C54	0.3 (5)
Cu1—P4—C15—C16	−117.7 (2)	C52—C53—C54—C55	0.7 (5)
C20—C15—C16—C17	−0.6 (5)	C53—C54—C55—C56	−1.2 (6)
P4—C15—C16—C17	173.9 (3)	C52—C51—C56—C55	0.3 (5)
C15—C16—C17—C18	1.0 (5)	P6—C51—C56—C55	−176.6 (3)
C16—C17—C18—C19	−0.2 (5)	C54—C55—C56—C51	0.7 (5)
C17—C18—C19—C20	−0.9 (5)	N8—P7—C57—C62	−174.0 (2)
C16—C15—C20—C19	−0.5 (5)	C63—P7—C57—C62	74.7 (3)
P4—C15—C20—C19	−175.2 (3)	S3—P7—C57—C62	−39.6 (3)
C18—C19—C20—C15	1.2 (5)	N8—P7—C57—C58	6.6 (3)
C9—P4—C21—C22	101.7 (3)	C63—P7—C57—C58	−104.7 (2)
C15—P4—C21—C22	−4.6 (4)	S3—P7—C57—C58	141.0 (2)
Cu1—P4—C21—C22	−128.0 (3)	C62—C57—C58—C59	−0.1 (5)
C9—P4—C21—C26	−80.7 (3)	P7—C57—C58—C59	179.3 (2)
C15—P4—C21—C26	173.0 (3)	C57—C58—C59—C60	−1.2 (5)
Cu1—P4—C21—C26	49.6 (3)	C58—C59—C60—C61	1.2 (5)
C26—C21—C22—C23	−2.3 (6)	C59—C60—C61—C62	0.1 (5)
P4—C21—C22—C23	175.3 (4)	C58—C57—C62—C61	1.4 (4)
C21—C22—C23—C24	0.7 (8)	P7—C57—C62—C61	−177.9 (2)
C22—C23—C24—C25	1.8 (7)	C60—C61—C62—C57	−1.5 (5)
C23—C24—C25—C26	−2.6 (6)	N8—P7—C63—C64	−137.6 (3)
C24—C25—C26—C21	0.9 (6)	C57—P7—C63—C64	−22.2 (3)
C22—C21—C26—C25	1.5 (5)	S3—P7—C63—C64	94.7 (3)
P4—C21—C26—C25	−176.3 (3)	N8—P7—C63—C68	44.5 (3)
C39—P5—C27—C32	98.5 (3)	C57—P7—C63—C68	159.9 (2)
C33—P5—C27—C32	−8.7 (3)	S3—P7—C63—C68	−83.2 (2)
Cu1—P5—C27—C32	−133.6 (2)	C68—C63—C64—C65	0.0 (5)
C39—P5—C27—C28	−75.8 (3)	P7—C63—C64—C65	−177.8 (2)
C33—P5—C27—C28	177.1 (3)	C63—C64—C65—C66	−0.1 (5)
Cu1—P5—C27—C28	52.2 (3)	C64—C65—C66—C67	0.1 (5)
C32—C27—C28—C29	0.0 (5)	C65—C66—C67—C68	−0.1 (5)
P5—C27—C28—C29	174.5 (3)	C66—C67—C68—C63	0.0 (5)
C27—C28—C29—C30	0.4 (6)	C64—C63—C68—C67	0.0 (5)
C28—C29—C30—C31	−0.1 (6)	P7—C63—C68—C67	178.0 (2)

### Hydrogen-bond geometry (Å, º)

**Table:**

*D*—H···*A*	*D*—H	H···*A*	*D*···*A*	*D*—H···*A*
C42—H42···C13^i^	0.95	2.91	3.586 (5)	129
C59—H59···C53^ii^	0.95	2.79	3.590 (5)	142
C56—H56···C60^iii^	0.95	2.69	3.589 (6)	157
C66—H66···C65^iv^	0.95	3.02	3.468 (5)	111
C55—H55···C55^v^	0.95	3.71	3.552 (5)	73
C55—H55···C56^v^	0.95	3.47	3.495 (5)	83

Symmetry codes: (i) −*x*+2, −*y*, −*z*+1; (ii) −*x*+1, −*y*+1, −*z*; (iii) *x*+1, *y*, *z*; (iv) −*x*+1, −*y*, −*z*; (v) −*x*+2, −*y*+1, −*z*.
